# Synthesis of Bi_2_S_3_/BiVO_4_ Heterojunction with a One-Step Hydrothermal Method Based on pH Control and the Evaluation of Visible-Light Photocatalytic Performance

**DOI:** 10.3390/ma10080891

**Published:** 2017-08-02

**Authors:** Deqiang Zhao, Wenwen Wang, Wenjuan Zong, Shimin Xiong, Qian Zhang, Fangying Ji, Xuan Xu

**Affiliations:** 1 Key Laboratory of Three Gorges Reservoir Region’s Eco-Environment Ministry of Education and National Centre for International Research of Low-Carbon and Green Buildings, Chongqing University, No. 174 Shazhengjie, Shapingba, Chongqing 400045, China; a2006silent@foxmail.com (D.Z.); zongwenjuan1992@163.com (W.Z.); 18512341504@163.com (S.X.); zhangqiancqu@126.com (Q.Z.); 2 National Centre for International Research of Low-Carbon and Green Buildings, Chongqing University, Chongqing 400045, China; 3 Faculty of Urban Construction and Environment Engineering, Chongqing 400045, China; www-vermeer@foxmail.com

**Keywords:** heterojunction, photocatalysis, hydrothermal method, bismuth vanadate, bismuth sulfide, pH value

## Abstract

The band gaps of bismuth vanadate (BiVO_4_) and bismuth sulfide (Bi_2_S_3_) are about 2.40 eV and 1.30 eV, respectively. Although both BiVO_4_ and Bi_2_S_3_ are capable of strong visible light absorption, electron–hole recombination occurs easily. To solve this problem, we designed a one-step hydrothermal method for synthesizing a Bismuth sulfide (Bi_2_S_3_)/Bismuth vanadate (BiVO_4_) heterojunction using polyvinylpyrrolidone K-30 (PVP) as a structure-directing agent, and 2-Amino-3-mercaptopropanoic acid (l-cysteine) as a sulfur source. The pH of the reaction solution was regulated to yield different products: when the pH was 7.5, only monoclinic BiVO_4_ was produced (sample 7.5); when the pH was 8.0 or 8.5, both Bi_2_S_3_ and BiVO_4_ were produced (samples 8.0 and 8.5); and when the pH was 9.0, only Bi_2_S_3_ was produced (sample 9.0). In sample 8.0, Bi_2_S_3_ and BiVO_4_ were closely integrated with each other, with Bi_2_S_3_ particles formed on the surface of concentric BiVO_4_ layers, but the two compounds grew separately in a pH solution of 8.5. Visible-light photocatalytic degradation experiments demonstrated that the degradation efficiency of the Bi_2_S_3_/BiVO_4_ heterojunction was highest when prepared under a pH of 8.0. The initial rhodamine B in the solution (5 mg/L) was completely degraded within three hours. Recycling experiments verified the high stability of Bi_2_S_3_/BiVO_4_. The synthesis method proposed in this paper is expected to enable large-scale and practical use of Bi_2_S_3_/BiVO_4_.

## 1. Introduction

Semiconductors such as bismuth oxybromide–bismuth oxyiodide [1], β-ZnMoO_4_ [2], metal–organic frameworks (MOFs) [3], and BiOxIy/GO [4] can facilitate the photodegradation of dyes. Bismuth oxides and their composites (e.g., bismuth oxyiodides [5], bismuth oxyiodide/graphitic carbon nitride [6], and BiO*_x_*I*_y_*/GO [7]) have been widely used for photocatalytic degradation of dyes, owing to their superior photocatalytic traits. Bismuth vanadate (BiVO_4_) has the advantages of no toxicity, low cost, high chemical stability, photocorrosion resistance, and strong response to visible light (*E*_g_ ~ 2.40 eV) [8]. However, because BiVO_4_ has a limited visible absorption range (<525 nm), and its electrons and holes recombine easily, the widespread application of this material is limited [9]. To improve the photocatalytic efficiency of BiVO_4_ under visible radiation, it is necessary to expand its visible absorption range, and limit the recombination of electrons and holes. On this basis, researchers have made considerable efforts towards BiVO_4_ modification. 

Doping is the most frequently used method for improving the visible absorption range of BiVO_4_. For instance, BiVO_4_ has been doped with rare earth metals (e.g., Bi_4_V_2−*x*_M*_x_*O_11−*x*_(M = Gd, Nd, Gd, M = Nd) [10]), europium [11], transition metals (e.g., Ag-doped BiVO_4_ [12] and Pd/BiVO_4_ [13]), and C [14,15]. These doping systems can effectively reduce the band gap of BiVO_4_ and increase its visible absorption range. However, these methods may elevate costs due to the use of precious metals, and increase heavy metal pollution. In addition, excessive doping may enhance electron–hole recombination.

A common method to restrain the recombination of electron–hole pairs is to construct a heterojunction. A number of studies have reported the synthesis of heterojunctions such as WO_3_/BiVO_4_ [16], CaFe_2_O_4_/BiVO_4_ [17], BiOCl/BiVO_4_ [18], and CO_3_O_4_/BiVO_4_ [19]. Although these heterojunctions effectively restrain the separation of electron–hole pairs, they do not have an expanded visible adsorption range.

Bismuth sulfide (Bi_2_S_3_) is a p-type semiconductor (*E*_g_ ~ 1.30 eV) [20] with a narrow band gap and a very strong response to visible light. The valence band (VB) position of Bi_2_S_3_ is more negative than that of BiVO_4_, whereas the conduction band (CB) position of Bi_2_S_3_ is more positive [21]. Therefore, Bi_2_S_3_ can be combined with n-type semiconductor BiVO_4_ to form a heterojunction with a strong capacity for visible light absorption. Using thioacetamide as a sulfur source, De-Kun Ma et al. [22] developed a two-step hydrothermal method to synthesize olive-shaped Bi_2_S_3_/BiVO_4_ microspheres with a limited chemical conversion route and enhanced photocatalytic activity. Canjun Liu et al. [23] used thiourea as a sulfur source in a two-step method for synthesizing Bi_2_S_3_ nanowires on BiVO_4_ nanostructures with enhanced photoelectrochemical performance. Xuehui Gao et al. [24] studied the enhanced photoactivity of mesoporous heterostructured Bi_2_S_3_/BiVO_4_ hollow discoid prepared using Na_2_S·9H_2_O as a sulfur source. 

In this work, we developed a simple one-step method to synthesize a Bi_2_S_3_/BiVO_4_ heterojunction using the amino acid l-cysteine as a sulfur source. We added the sulfur source during the preparation of BiVO_4_ using a one-step hydrothermal method, and then regulated the pH of the system to cause Bi_2_S_3_ to form on the BiVO_4_, thereby creating the Bi_2_S_3_/BiVO_4_ heterojunction. We found that this complex material demonstrated a strong capacity for visible light absorption and separation of electron–hole pairs. The use of the heterojunction as a catalyst for rhodamine B (RhB) degradation was investigated under visible light irradiation, and possible formation and degradation mechanisms strongly affected by pH were proposed. The results indicate that this complex material has potential for use in environmental and optoelectronic applications.

## 2. Materials and Methods

### 2.1. Materials and Reagents

The materials included analytical grade pure bismuth nitrate (Bi(NO_3_)_3_·5H_2_O (Chengdu Industrial Development Zone Xinde Mulan, Chengdu, China), analytical grade pure sodium hydroxide powder (NaOH, Chongqing Chuandong Chemical Company, Chongqing, China), analytical grade polyvinylpyrrolidone K-30 (PVP) (Chengdu Kelong Chemical Co., Ltd., Chengdu, China), ammonium metavanadate (NH_4_VO_3_, Chongqing Chuandong Chemical Company, Chongqing, China), l-cysteine (Aladdin Industrial Corporation, Shanghai, China), rhodamine B (RhB, Tianjin Guangfu Fine Chemical Research Institute, Tianjin, China), nitric acid (HNO_3_, Chengdu Area of the Industrial Development Zone Xinde Mulan, Chengdu, China), ethylene glycol, (C_4_H_4_O_6_KNa_4_·H_2_O), silver nitrate (AgNO_3_), p-benzoquinone (PBQ), and isopropanol (IPA) (Chongqing Chuandong Chemical Company, Chongqing Chuandong, China).

### 2.2. Synthesis of Photocatalysts

The pH of the reaction solution was set to 7.5, 8.0, 8.5, or 9.0 (Sample-7.5, Sample-8.0, Sample-8.5, and Sample-9.0, respectively). During synthesis, 2 mmol Bi(NO_3_)·5H_2_O and 0.6 mmol l-cysteine were dissolved in 4 mL of 4 mol/L HNO_3_. Then, 50 mL of deionized water was added to the solution, and the mixture was stirred for 30 min to obtain solution A. Similarly, 2 mmol NH4VO_3_ was dissolved in 4 mL of 2 mol/L NaOH, and the mixture was stirred for 30 min to obtain solution B. Solutions A and B were mixed together, and the pH was adjusted to 7.5, 8.0, 8.5, or 9.0 using NaOH or HNO_3_. The solutions were transferred into a 100-mL, Teflon-lined autoclave, heated at 180 °C for 16 h, and allowed to cool to room temperature. Finally, the solutions were centrifuged to obtain the final products. The samples were rinsed with distilled water and ethanol six times, and the pH of the filtrate was measured after the last rinse. If the pH of a sample was not neutral, the sample was further rinsed until a pH of ~7 was obtained. Then, the products were dried at 60 °C for 12 h in vacuum.

### 2.3. Characterization

The crystal structures of prepared samples were characterized using X-ray diffraction (XRD) under CuK radiation with a Rigaku D/Max2500pc (Tokyo, Japan) diffractometer (scanning angle 2θ from 10° to 70°, and scanning rate of 4°/min). A high-voltage (10 kV) Tescan FEG-SEM microscope (TESCAN, MARI3, Brno, Czech Republic) was used to acquire scanning electron microscopy (SEM) images of the prepared samples, and the elemental composition was characterized with an energy dispersive X-ray detector (EDX). A JEM-3010 electron microscope (JEOL, Tokyo, Japan) was used to perform transmission electron microscopy (TEM) at an acceleration voltage of 300 kV. Fourier transform infrared (FT-IR) spectra of prepared samples were recorded on a Shimadzu IR Prestige-1 spectrometer (Tokyo, Japan) using the KBr pellet technique. The chemical characteristics of sample surfaces were investigated using X-ray photoelectron spectra (XPS) acquired with a PHI5000 (ULVAC-PHI,INC., Kanagawa Prefecture, Japan) versa probe system under monochromatic Al K X-rays. A Hitachi U-3010 UV-Vis spectrophotometer (Tokyo, Japan) was used to perform UV-Vis diffuse reflectance spectroscopy (UV-Vis DRS) using the “Abs” data mode. The photoluminescence (PL) spectra of the photocatalysts were obtained using a Hitachi F-7000 (Tokyo, Japan) spectrometer with an excitation wavelength of 280 nm. A CHI Electrochemical Workstation (CHI 760E, Shanghai Chenhua Co., Ltd., Shanghai, China) was used to evaluate the photoelectrochemical properties of the samples. The visible light source was a 500 W Xe lamp (with a <400 nm UV cutoff filter), and all experiments were carried out at room temperature. 

### 2.4. Evaluation of Photocatalytic Activity

The photocatalytic activity of samples was evaluated under visible radiation at room temperature by measuring the degradation rate of RhB. For the experiments, 0.20 g of catalyst was added to 200 mL of 5 mg/L RhB aqueous solution, and the mixture was stirred for 30 min in the dark to achieve adsorption–desorption equilibrium between the dye and the catalyst. The experimental solution was placed 350 mm from the 500-W Xe lamp (with a <400 nm UV cutoff filter). Samples were collected after every 0.5 h of irradiation, and then centrifuged (10,000 r/min) to remove the catalyst. Subsequently, the absorbance of the solution at 552 nm was measured to indicate the concentration of the remaining dye. For comparison, the photocatalytic experiments were conducted with Samples 7.5, 8.0, 8.5, and 9.0 as catalysts, and with no catalyst.

## 3. Results and Discussion

### 3.1. XRD Pattern Analysis

**Figure 1:**
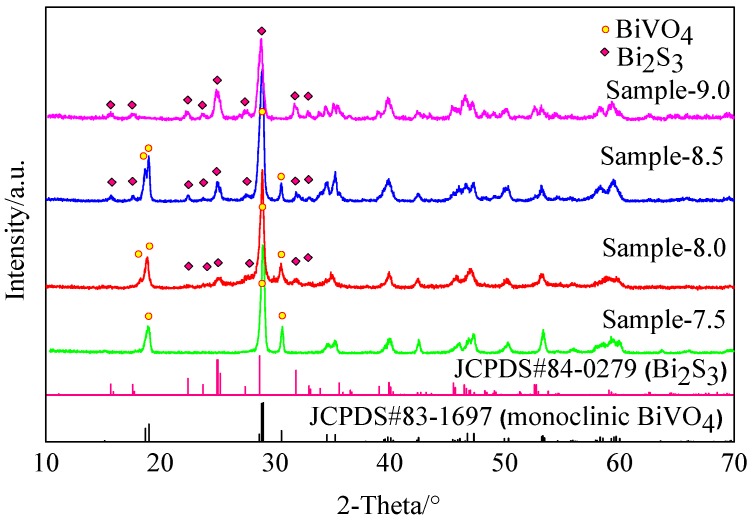
X-ray diffraction (XRD) patterns.

When the synthesis reaction was performed in a pH solution of 7.5, the XRD diffraction pattern of the resulting sample was similar to that of monoclinic BiVO_4_ (yellow line in Figure 1 JCPDS#83-1697) [25], indicating that the sample was monoclinic BiVO_4_. In contrast, the XRD diffraction peaks of samples 8.0 and 8.5 showed characteristics of both BiVO_4_ and Bi_2_S_3_ (JCPDS#84-0279) [26]. As the pH increased, the Bi_2_S_3_ peak increased in both height and intensity. However, when the pH of the precursor solution was 9.0, only Bi_2_S_3_ was obtained. Therefore, the SEM, XPS, and TEM results indicate that a Bi_2_S_3_/BiVO_4_ heterojunction may be synthesized when the reaction was performed under a pH of 8.0 or (and) 8.5. 

### 3.2. Morphology Analysis Based on SEM, HRTEM, and FT-IR

**Figure 2:**
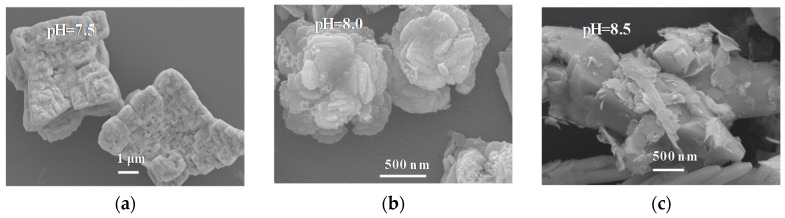

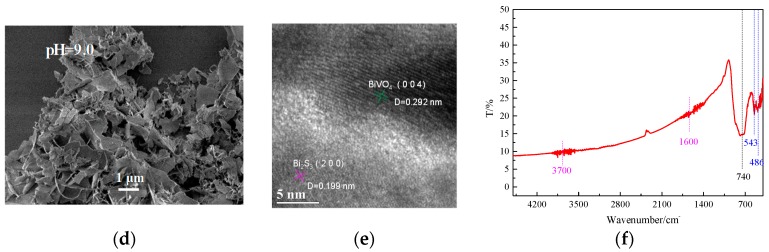
Scanning electron microscopy (SEM) (**a**–**d**) of samples, HRTEM (**e**) of Sample-8.0, and Fourier–transform infrared (FT-IR) spectra (**f**) of Sample-8.0.

According to the SEM images (Figure 2), sample 7.5 was cube-like in shape, with a length of about 5 μm, whereas sample 8.0 consisted of concentric layers, with some Bi_2_S_3_ particles dotting the outer layers. Sample 8.5 mainly consisted of BiVO_4_ shaped as hexagonal pyramids and some flakes of Bi_2_S_3_. Sample 9.0 consisted of only Bi_2_S_3_ flakes. Therefore, the pH of the precursor solution strongly influenced the prepared products. Characteristic stripes of BiVO_4_ (004) [27] and Bi_2_S_3_ (200) [28] can be observed from the HRTEM results (Figure 2e). Figure 2f shows the FT-IR spectra of sample 8.0. The broad absorptions at 740 cm^−1^ can be attributed to BiVO_4_ [29]. Furthermore, absorption at 543 cm^−1^ and 486 cm^−1^ can be ascribed to Bi_2_S_3_ [30], whereas the broad and weak peaks around 3700 cm^−1^ and 1600 cm^−1^ are due to the vibrational rotation and bending deformation of very small amounts of water molecules in the sample [31]. These results show that the heterojunction was successfully prepared when the pH of precursor solution was 8.0.

### 3.3. Analysis of Composition and Chemical States Based on EDX and XPS

**Figure 3:**
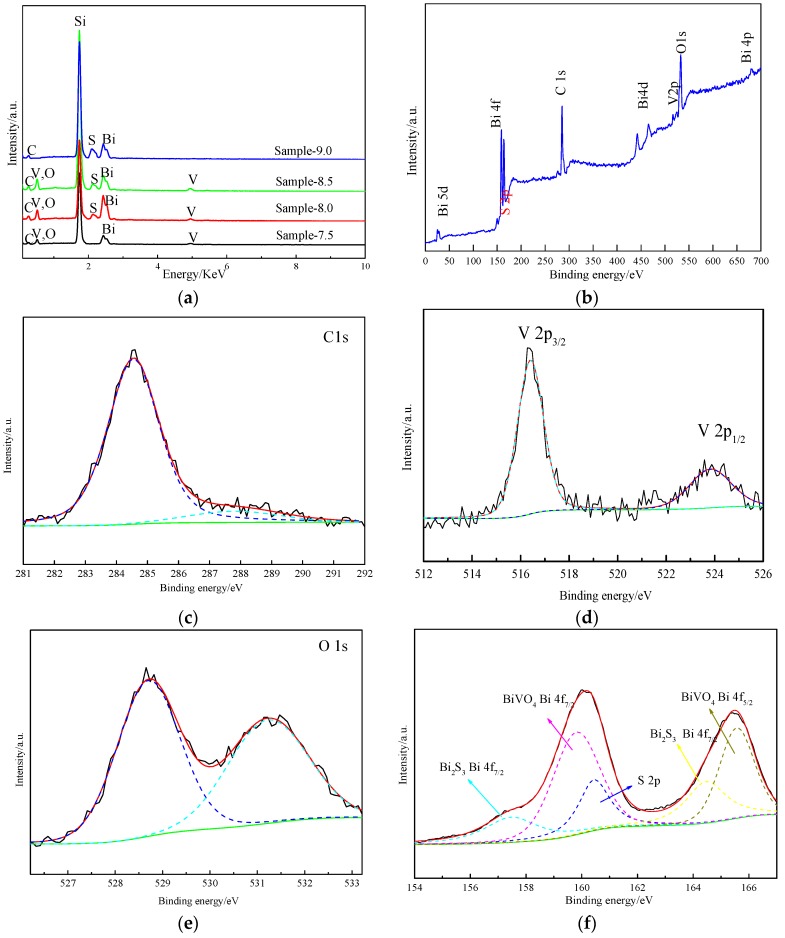
Energy dispersive X-ray detector (EDX) spectra of all samples (**a**) and X-ray photoelectron spectra (XPS) spectra of Sample-8.0 (**b**–**f**): (**b**) survey XPS spectrum, (**c**) C 1*s* peaks, (**d**) V 2*p*3/2 and V 2*p*1/2 peaks, (**e**) O 1*s* peak, and (**f**) Bi 4*f*5/2, Bi 4*f*7/2 and S 2*p* peaks.

The composition and chemical states of samples were analyzed based on EDX and XPS. According to the EDX patterns of samples (Figure 3a), sample 7.5 contained the elements C, Bi, O, and V. When the pH of the precursor solution was 8.0 and 8.5, the sample contained C, Bi, O, V, and S. As the pH increased to 9.0, only C, Bi, and S were tested. Carbon was mostly due to CO_2_ adsorption from the atmosphere [32], whereas Si was from the substrate. The wt % of Bi, V, O, S, and C in the samples tested via EDX are listed in Table 1, as well as the speculated composition. The molar ratio of BiVO_4_:Bi_2_S_3_ was about 7.42:1 and 6.07:1 in samples 8.0 and 8.5, respectively.

The chemical state of Sample-8 was analyzed by XPS. Figure 3b shows fully scanned spectra and the S 2*s* orbital of Sample-8.0 within the range of 0–700 eV. As shown in the spectra, the composite material was composed of Bi, O, V, C, and S. The binding energy of C l*s* of the non-oxygenated ring C was corrected to 284.6 eV (Figure 3c) [33]. The V 2*p* core level spectrum in Figure 3d indicates that the binding energies (516.8 and 524.4 eV) for V 2*p* are in accordance with former reports on V^5+^ in BiVO_4_ [34]. The XPS signals of O 1*s* are 529.7 eV and 532.7 eV (Figure 3e) [14], respectively; this can be explained by the existence of O_2_^−^ anions in BiVO_4_. According to Figure 3f, the peaks with binding energies of 159.3 eV and 164.6 eV are respectively related to Bi 4*f*7/2 and Bi 4*f*5/2 in BiVO_4_ [35]. The peaks with binding energies of 157.2 eV and 162.6 eV respectively correspond to Bi 4*f*7/2 and Bi 4*f*5/2 in Bi_2_S_3_ [36]. The peak with binding energy of 160.4 eV corresponds to the S 2*p* transition [37]. It is known that the binding energy of pure Bi_2_S_3_ is 158.9 ± 0.1 eV, whereas the measured value is 158.4 ± 0.1 eV [38]. This shift indicates the interfacial chemical interaction between Bi_2_S_3_ and BiVO_4_ [39]. In conclusion, the XRD, SEM, TEM, EDX, and XPS results indicate that Bi_2_S_3_ quantum dots successfully formed on BiVO_4_.

### 3.4. Optical Properties Characterized by UV-Vis DRS, Transient Photocurrent Response, and PL 

**Figure 4:**
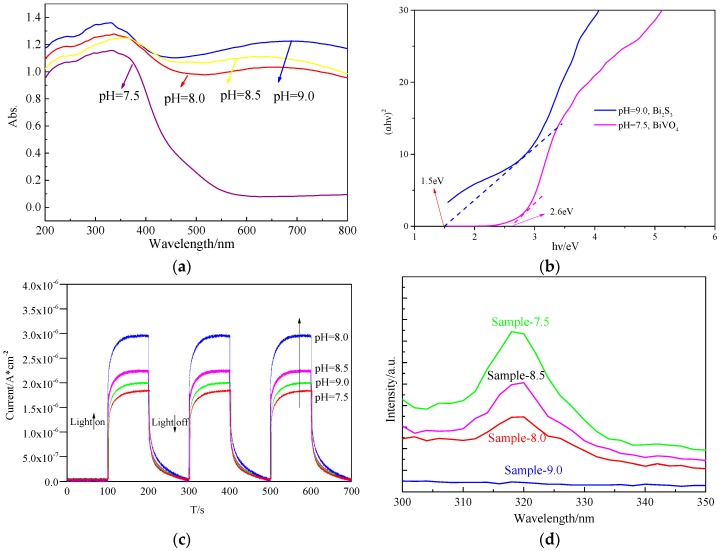
(**a**) UV-Vis diffuse reflectance spectra of samples; (**b**) Estimated band gaps of UV-Vis spectra of the samples; (**c**) Transient photocurrent response; (**d**) Photoluminescence (PL) spectra at an excitation wavelength of 280 nm.

Both BiVO_4_ [40,41] and Bi_2_S_3_ [22,42,43,44] display characteristics of direct transition. The light absorption properties and the band gap of the semiconductor can be determined based on UV-Vis absorption spectra (Figure 4a). Moreover, the band gap can be obtained from the slope of (Ahυ)^2^ versus hυ using Equation (1):

(1) $$\mathrm{Ah}\nu \;=\;C{\left(h\nu \;-\;E_{g}\right)}^{1/2}$$

(2) $$E_{VB}\;=\;\chi \;-\;E_{e}\;+\;0.5\;E_{g}$$

(3) $$E_{CB}\;=E_{VB}\;-\;E_{g}$$

where A is the absorption coefficient, *E_g_* is the band gap energy, h is Planck’s constant, ν is the incident light frequency, and C is a constant.

According to Figure 4b, sample 7.5 contained BiVO_4_. Most of the absorption regions were concentrated in the ultraviolet region, with a few concentrated in the visible region, and the band gap was 2.6 eV. Sample 9.0 contained Bi_2_S_3_, and the spectra show almost complete absorption, with a band gap of about 1.5 eV. Based on Equations (2) and (3), the VB values of Bi_2_S_3_ and BiVO_4_ are 1.52 and 2.84 eV, respectively, and the CB values of Bi_2_S_3_ and BiVO_4_ are 0.02 and 0.24 eV, respectively. Thus, Bi_2_S_3_ enhanced the absorption of visible light by BiVO_4_.

Photoelectrochemical tests were conducted to analyze the excitation, separation, and transfer of carriers in the catalyst. Photoanodes were prepared by electrophoretic deposition on ITO glass supports. The electrophoretic deposition was carried out in an acetone solution (20 mL) containing iodine (50 mg) and photocatalyst powder (50 mg), and then dispersed via sonication for 5 min. The ITO electrode (1.0 × 2.0 cm^2^ was immersed in the solution with a Pt electrode, and a 30 V differential was applied for 100 s using a potentiostat. After this process was repeated twenty times, the electrode was dried and calcined at 200 °C for 2 h. The exposed effective area of the ITO glass was controlled to be 1.0 × 1.0 cm^2^. According to the transient photocurrent responses of the samples under visible irradiation (Figure 4c), the photocurrent of sample 8.0 had the highest density, followed by sample 8.5, sample 9.0, and finally sample 7.5. The PL spectra (excitation wavelength of 280 nm) are shown in Figure 4d. The characteristic peak of bismuth vanadate is visible at 320 nm [45]. No peak was visible in sample 9.0 owing to the presence of bismuth sulfide. In contrast, sample 7.5 had the strongest peak, followed by sample 8.5, and sample 8.0. This is because sample 8.0 contained a Bi_2_S_3_/BiVO_4_ heterojunction, which is conductive to the separation of electron–hole pairs. Sample 8.5 was produced by physical contact of Bi_2_S_3_ and BiVO_4_. Sample 9.0 contained Bi_2_S_3_, but even though this catalyst can almost completely absorb visible light, the recombination of electrons and holes may occur due to the narrow band gap. Sample 7.5 contained BiVO_4_, which has a limited visible absorption range. Therefore, sample 8.0 displays the strongest potential for use in photoelectric and environmental applications.

### 3.5. Evaluation of Photocatalytic Activity Based on RhB Degradation

**Figure 5:**
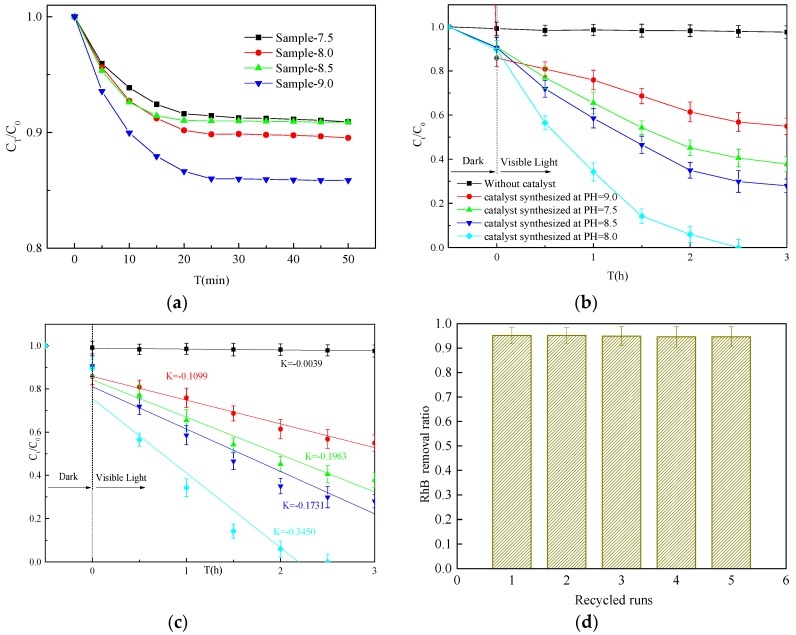

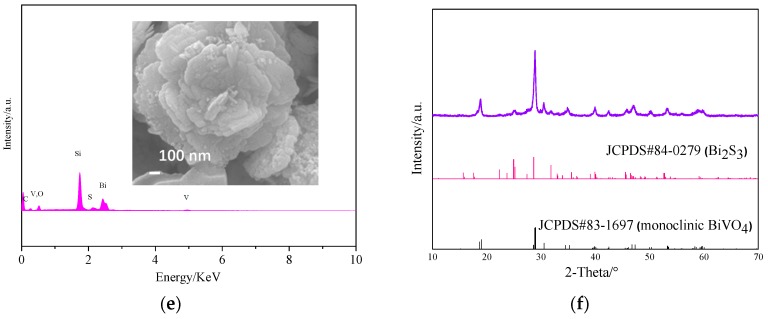
(**a**) Adsorption–desorption equilibrium test results; (**b**) Degradation of rhodamine B (RhB) using different catalysts under visible light irradiation; (**c**) Linear fit of the photocatalytic reaction and the reaction rate constant k; (**d**) Photocatalytic degradation of RhB reusing photocatalyst sample 8 after filtration; (**e**) EDX and SEM (inset) of sample 8 after recycling five times; (**f**) XRD of sample 8 after recycling five times.

Figure 5a shows the results of the adsorption–desorption equilibrium tests. The samples were collected once every 5 min during the dark reaction stage. The RhB concentrations of the various solutions stabilized over time. Dyes and catalysts reached adsorption–desorption equilibrium after stirring for 30 min in the dark. Figure 5b shows the photocatalytic activities of the samples under visible irradiation for 3 h, and the corresponding degradation kinetics constants are shown in Figure 5c. For comparison, the RhB degradation rate was also estimated with no catalyst. The results indicate that 45.07%, 62.13%, 72.00%, and 100.00% of the RhB was degraded when samples 9.0, 7.5, 8.5, and 8.0 were used as catalysts, respectively. This indicates that sample 8.0 has the highest catalytic activity, followed by sample 8.5, sample 7.5, and sample 9.0. Sample 8.0 can be easily recycled by simple filtration, and its degradation effect was respectively maintained at 95.19%, 95.18%, 94.91%, 94.64%, and 94.63% after five recycling cycles (Figure 5d). The EDX, SEM, and XRD results for sample 8.0 after five recycling cycles indicated that no leaching of elements occurred and the morphology remained unchanged (Figure 5e,f). This indicated that sample 8.0 has an excellent heterostructure. Furthermore, the Bi_2_S_3_ dots were very small (19.7 ± 2.2 nm, calculated based on the Sherer relation); thus, the specific surface area of the sample was larger than that of pure BiVO_4_ (Table 2). The specific surface area of samples 7.5, 8.0, 8.5, and 9.0 was 6.423, 19.527, 12.642, and 21.165 m^2^ g^−1^, respectively, indicating that Bi_2_S_3_ greatly impacted the specific surface area of the samples. The formation of the heterojunction, and the greater specific surface area of BiVO_4_ of sample 8.0 improved its photocatalytic activity. These features are very important for its practical application and modification.

### 3.6. Postulated Heterojunction Formation Mechanism

The pH of the reaction system had a strong influence on the crystal phase and morphology of the synthesized products [46]. By fixing the amounts of materials and l-cysteine, as well as the reaction parameters (except pH), the synthesis reaction was conducted under varying pH values. To explain the results, we speculated a series of reactions that can occur successively based on the amount of OH^−^. First, when Bi^3+^ is added to the l-cysteine system, chelation between the metal and amino acids can occur (4) [47].

(4) $${\mathrm{Bi}}^{3+}+nL-\mathrm{cysteine}\to {\left[\mathrm{Bi}\left(L-\mathrm{cysteine}\right)n\right]}^{3+}$$

Below, we discuss what happens when the pH of different systems is adjusted. 

The pH was lowest in the first system. Pure monoclinic BiVO_4_ was produced. The probably reaction is the (5), because we know that S^3−^ did not exist in the system and S^3−^ can exchange ions with BiVO_4_ to form Bi_2_S_3_ [32].

(5) $${\left[\mathrm{Bi}\left(L-\mathrm{cysteine}\right)n\right]}^{3+}+{\mathrm{VO}}_{3}^{-}+H^{+}\to {\mathrm{BiVO}}_{4}↓+H_{2}S↑+{\;H}_{2}O$$

In the second system, the pH was adjusted to 8.0, and the composite Bi_2_S_3_/BiVO_4_ was produced. We can see that some Bi_2_S_3_ formed on the BiVO_4_; thus, we speculate that (5) still took place in the system. Since OH^−^ remains in the system, (6) will occur, forming S^2−^. Then, as previously mentioned, S^2−^ will react with BiVO_4_ to form Bi_2_S_3_. As the amount of S in the system was low (0.6 mmol in total), some of the BiVO_4_ formed Bi_2_S_3_ as shown in Figure 2b.

(6) $$H_{2}S+{\mathrm{OH}}^{-}\to S^{2-}+{\;H}_{2}O\;$$

(7) $${\mathrm{BiVO}}_{4}+S^{2-}\to {\mathrm{Bi}}_{2}S_{3}↓+{\mathrm{VO}}_{4}^{+}$$

As the pH increased to 8.5, the first reaction could no longer take place. Instead, (8) occurred, causing the OH^−^ concentration to decrease, followed by (9). Sample 8.5 consisted of both Bi_2_S_3_ and BiVO_4_, separately.

(8) $${\left[\mathrm{Bi}\left(L-\mathrm{cysteine}\right)n\right]}^{3+}+{\mathrm{NH}}_{4}^{+}+{\mathrm{OH}}^{-}\to {\mathrm{Bi}}_{2}S_{3}↓+{\mathrm{NH}}_{3}↑+{\;H}_{2}O\;$$

(9) $${\mathrm{Bi}}^{3+}+{\mathrm{VO}}_{3}^{-}\to {\mathrm{BiVO}}_{4}↓\;$$

Equation (8) could still take place under a pH of 9.0. The synthesized sample contained only Bi_2_S_3_ due to the high OH^−^ concentration. The remaining Bi^3+^ first reacted with OH^−^ to form Bi(OH)_3_, as shown in (10). The reaction then continued, causing Bi(OH)_3_ to dissolve, and forming Bi(OH)_4_^−^ (11) [48].

(10) $${\mathrm{Bi}}^{3+}+{\mathrm{OH}}^{-}\to \mathrm{Bi}{\left(\mathrm{OH}\right)}_{3}↓$$

(11) $$\mathrm{Bi}{\left(\mathrm{OH}\right)}_{3}+{\mathrm{OH}}^{-}\to \mathrm{Bi}{\left(\mathrm{OH}\right)}_{4}^{-}$$

### 3.7. Postulated Degradation Mechanism

**Figure 6:**
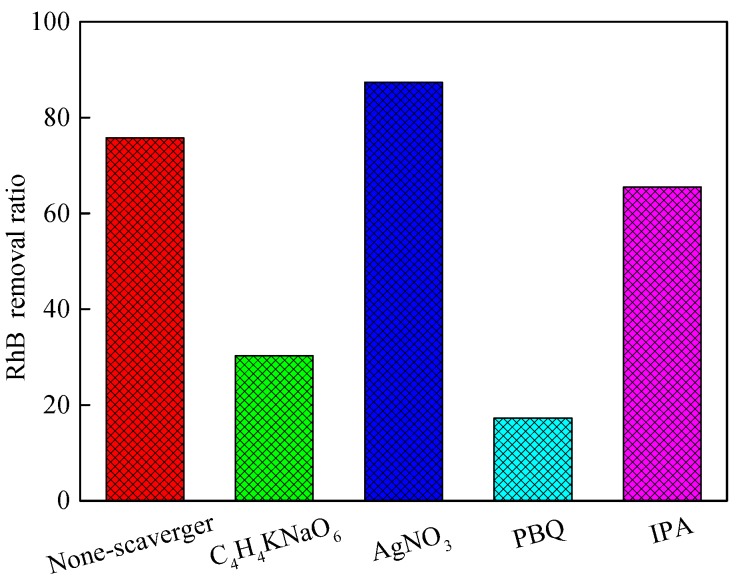
Scavenger effect of sample 8 photocatalyst.

Active species such as ·O_2_^−^, ·OH, e^−^, and holes(h^+^)play an important role in the photodegradation of dyes [49]. To investigate the photocatalytic mechanism and activity of Bi_2_S_3_/BiVO_4_, the contribution of each active species to the photodegradation performance was examined based on a scavenger experiment [50]. In this experiment, potassium sodium tartrate (C_4_H_4_O_6_KNa·4H_2_O, 0.1 mmol) was used as the h^+^ scavenger, silver nitrate (AgNO_3_, 0.1 mmol) was used as the e^−^ scavenger, p-benzoquinone (PBQ, 0.1 mmol) was used as the ·O_2_^−^ scavenger, and isopropanol (IPA, 0.1 mmol) was used as the ·OH scavenger. These scavengers were added to RhB solutions containing Bi_2_S_3_/BiVO_4_, and the solutions were irradiated with visible light for 1.5 h. According to Figure 6, the addition of the h^+^ scavenger (C_4_H_4_O_6_KNa) and the ·O_2_^−^ scavenger (IPA) severely suppressed the catalytic performance, suggesting that h^+^ and ·O_2_^−^ are the main reactive species contributing to the photodegradation of RhB. After the addition of the ·OH scavenger (IPA), the catalytic degradation effect was slightly inhibited, which suggests that ·OH plays an insignificant role in this reaction system. The ·OH was mainly produced by oxidation of H_2_O to ·O_2_^−^ and OH^−^ through BiVO_4_ VB [1]. Lizhen Ren et al. reported that the VB of Bi_2_S_3_ was not sufficient to oxidize OH^−^ to ·OH [51]. After adding the e^−^ scavenger (AgNO_3_), the degradation efficiency increased owing to e^−^ consumption and increased h^+^ efficiency; therefore, h^+^ plays the most important role in this degradation process and can directly degrade dyes [1,49].

The scavenger experiments confirmed the existence of ·O_2_^−^, ·OH, e^−^, and h^+^ in the catalytic system. A thorough understanding of the photocatalytic degradation mechanism is necessary for the actual application of Bi_2_S_3_/BiVO_4_ heterojunctions. The postulated degradation mechanism is shown in Schematic 1. Figure 5a demonstrates that the concentration of the dye did not significantly change without the addition of a catalyst. In contrast, photocatalyst addition significantly reduced the dye concentration. The adsorption of dye onto the catalyst surface is the primary process, as shown in (12). RhB is sensitive to visible light, as indicated by (13) [52]. The potential energy of the RhB LUMO and HOMO was −1.42 and 0.95 eV, respectively [53]. The VB values of Bi_2_S_3_ and BiVO_4_ were 1.52 and 2.84 eV, respectively, and the CB values of Bi_2_S_3_ and BiVO_4_ were 0.02 and 0.24 eV, respectively. Hence, the photosensitive dye transferred electrons to the CB of semiconductors (14) [54]. BiVO_4_ and Bi_2_S_3_ were excited under visible radiation, causing transfer of Bi_2_S_3_ and BiVO_4_ electrons from VB to CB, and leaving holes in VB ((15) and (16)). Due to the formation of the heterojunction, the holes in the VB of BiVO_4_ shifted to the VB of Bi_2_S_3_, while the electrons in CB of Bi_2_S_3_ shifted to the CB of BiVO_4_. Thus, the carriers of the electron–hole pair were separated ((17) and (18)). H_2_O can ionize OH, as shown (19). The photogenerated electrons can react with dissolved oxygen molecules (O_2_) to yield superoxide radical anions (·O_2_^−^), as shown in (20), and ·O_2_^−^ can react with H_2_O to produce ·OH (21) [55,56]. As previously mentioned, the VB of BiVO_4_ can oxidize OH^−^ to ·OH (22), and h^+^, ·O_2_^−^, and ·OH are strong oxidizing agents for the decomposition of organic dyes (23) [57]. The whole set of redox reactions can be summarized as follows:

(12) $${\mathrm{BiVO}}_{4}/{\mathrm{Bi}}_{2}S_{3}+\mathrm{RhB}\;\to \mathrm{adsorption}$$

(13) $$\mathrm{RhB}+\;h\nu \to {\mathrm{RhB}}^{*}$$

(14) $${\mathrm{RhB}}^{*}\;+{\mathrm{BiVO}}_{4}/{\mathrm{Bi}}_{2}S_{3}\;\to {\mathrm{BiVO}}_{4}/{\mathrm{Bi}}_{2}S_{3}\;\left(e^{-}\right)+{\;\mathrm{RhB}}^{+}$$

(15) $${\mathrm{Bi}}_{2}S_{3}+\;h\nu \to {\mathrm{Bi}}_{2}S_{3}\;\left(h^{+}\right)+{\;\mathrm{Bi}}_{2}S_{3}\;\left(e^{-}\right)$$

(16) $${\mathrm{BiVO}}_{4}+\;h\nu \to {\;\mathrm{BiVO}}_{4}\;\left(h^{+}\right)+{\;\mathrm{BiVO}}_{4}\;\left(e^{-}\right)$$

(17) $${\mathrm{Bi}}_{2}S_{3}\;\left(e^{-}\right)\;+{\;\mathrm{BiVO}}_{4}\to {\mathrm{BiVO}}_{4}\;\left(e^{-}\right)$$

(18) $${\mathrm{BiVO}}_{4}\;\left(h^{+}\right)+{\;\mathrm{Bi}}_{2}S_{3}\to {\mathrm{Bi}}_{2}S_{3}\;\left(h^{+}\right)$$

(19) $$H_{2}O\to H^{+}\;+{\mathrm{OH}}^{-}$$

(20) $$\left(e^{-}\right)+{\;O}_{2}\to \;\cdot O_{2}{\;}^{-}$$

(21) $$H_{2}O+\cdot O_{2}{\;}^{-}\to \cdot \mathrm{OH}\;+{\mathrm{OH}}^{-}+O_{2}$$

(22) $${\mathrm{BiVO}}_{4}\;\left(h^{+}\right)+{\mathrm{OH}}^{-}\to \cdot \mathrm{OH}$$

(23) $$\left(h^{+}\right)\;\mathrm{or}\;\cdot O_{2}{\;}^{-}\mathrm{or}\;\cdot \mathrm{OH}+{\;\mathrm{RhB}\;\mathrm{or}\;\mathrm{RhB}}^{+\;}\;\to \;\mathrm{degradation}\;\mathrm{products}$$

## 4. Conclusions

A one-step method for synthesizing a Bi_2_S_3_/BiVO_4_ heterojunction with high photocatalytic activity under visible irradiation was developed based on pH regulation of the reaction solution. The morphologies and compositions of prepared samples were characterized with various analytical methods. The degradation rate of RhB under visible irradiation was used to estimate the photocatalytic activity of prepared samples. The photocatalytic activity of sample 8.0 was higher than that of samples 7.5, 8.5, and 9.0, because the former contained a heterojunction.

## Figures and Tables

**Schematic 1:**
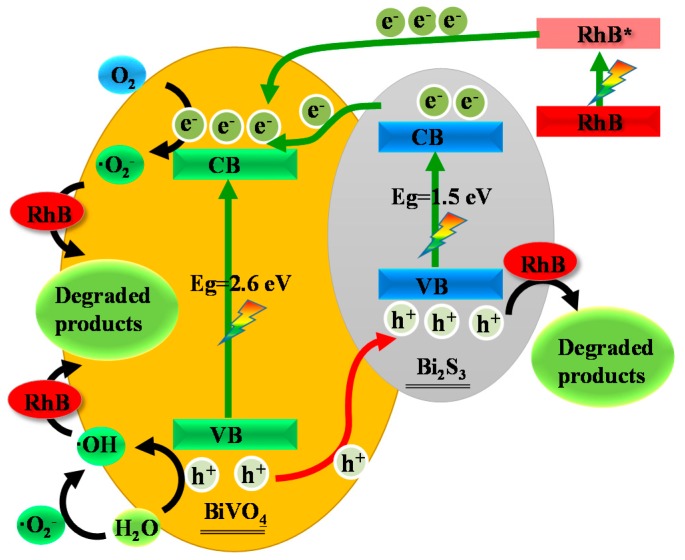
Postulated mechanism for RhB degradation catalyzed with sample 8.0.

**Table 1:** Sample composition based on EDX.

Sample	Bi	V	O	S	C	Molar Ratio
	wt %	wt %	wt %	wt %	wt %	BiVO_4_:Bi_2_S_3_
Sample-7.5	61.36	15.89	18.94	0.00	3.81	/
Sample-8.0	76.04	14.50	2.26	3.69	3.51	7.42:1
Sample-8.5	79.76	11.64	2.09	3.62	2.89	6.07:1
Sample-9.0	78.75	0.00	0.00	17.67	3.58	/

**Table 2:** Brunner−Emmet−Teller (BET) measurements of samples.

Sample	Mean Pore Size (nm)	Pore Volume (cm^3^ g^−1^)	Specific Surface Area (m^2^ g^−1^)
Sample-7.5	15.765	0.024	6.423
Sample-8.0	15.462	0.021	19.527
Sample-8.5	14.459	0.018	12.642
Sample-9.0	17.265	0.030	21.165

## References

[1] Jiang Y.R., Chou S.Y., Chang J.L., Huang S.T., Lin H.P., Chen C.C. (2015). Hydrothermal synthesis of bismuth oxybromide-bismuth oxyiodide composites with high visible light photocatalytic performance for the degradation of CV and phenol. RSC Adv..

[2] Jiang Y.R., Lee W.W., Chen K.T., Wang M.C., Chang K.H., Chen C.C. (2014). Hydrothermal synthesis of β-ZnMoO_4_ crystals and their photocatalytic degradation of Victoria Blue R and phenol. J. Taiwan Inst. Chem. Eng..

[3] Zubair H., Sung Hwa J. (2015). Removal of hazardous organics from water using metal-organic frameworks (MOFs): Plausible mechanisms for selective adsorptions. J. Hazard. Mater..

[4] Chou S.Y., Chung W.H., Chen L.W., Dai Y.M., Lin W.Y., Lin J.H., Chen C.C. (2016). A series of BiOxIy/GO photocatalysts: Synthesis, characterization, activity, and mechanism. RSC Adv..

[5] Lee W.W., Lu C.S., Chuang C.W., Chen Y.J., Fu J.Y., Siao C.W., Chen C.C. (2015). Synthesis of Bismuth Oxyiodides and Their Composites: Characterization, Photocatalytic Activity, and Degraded Mechanisms. RSC Adv..

[6] Chou S.Y., Chen C.C., Chen L.W., Dai Y.M., Lin J.H., Lee W.W. (2016). Novel synthesis of bismuth oxyiodide/graphitic carbon nitride nanocomposite with enhanced visible-light photocatalytic activity. RSC Adv..

[7] Lee Y.H., Dai Y.M., Fu J.Y., Chen C.C. (2017). A series of bismuth-oxychloride/bismuth-oxyiodide/graphene-oxide nanocomposites: Synthesis, characterization, and photcatalytic activity and mechanism. Mol. Catal..

[8] Liu S., Tang H., Zhou H., Dai G., Wang W. (2016). Photocatalytic perfermance of sandwich-like BiVO_4_ sheets by microwave assisted synthesis. Appl. Surf. Sci..

[9] Xiao B.C., Lin L.Y., Hong J.Y., Lin H.S., Song Y.T. (2017). Synthesis of a monoclinic BiVO_4_ nanorod array as the photocatalyst for efficient photoelectrochemical water oxidation. RSC Adv..

[10] Lee C.K., Ong C.S. (1999). Synthesis and characterisation of rare earth substituted bismuth vanadate solid electrolytes. Solid State Ion..

[11] Zhang A., Zhang J. (2010). Effects of europium doping on the photocatalytic behavior of BiVO_4_. J. Hazard. Mater..

[12] Xu X., Du M., Chen T., Xiong S., Wu T., Zhao D., Fan Z. (2016). New insights into Ag-doped BiVO_4_ microspheres as visible light photocatalysts. RSC Adv..

[13] Lei G. (2008). Novel Pd/BiVO_4_ composite photocatalysts for efficient degradation of methyl orange under visible light irradiation. Mater. Chem. Phys..

[14] Zhao D., Zong W., Fan Z., Xiong S., Du M., Wu T., Fang Y.W., Ji F., Xu X. (2016). Synthesis of Carbon Doped BiVO_4_@Multi-walled Carbon Nanotubes with High Visible Light Absorption Behavior and Evaluation of Its Photocatalytic Property. CrystEngComm.

[15] Yin C., Zhu S., Chen Z., Zhang W., Gu J., Zhang D. (2013). One step fabrication of C-doped BiVO_4_ with hierarchical structures for a high-performance photocatalyst under visible light irradiation. J. Mater. Chem. A.

[16] Su J., Guo L., Bao N., Grimes C.A. (2011). Nanostructured WO_3_/BiVO_4_ Heterojunction Films for Efficient Photoelectrochemical Water Splitting. Nano Lett..

[17] Kim E.S., Kang H.J., Magesh G., Kim J.Y., Jang J.W., Lee J.S. (2014). Improved photoelectrochemical activity of CaFe2O_4_/BiVO_4_ heterojunction photoanode by reduced surface recombination in solar water oxidation. ACS Appl. Mater. Interfaces.

[18] He Z., Shi Y., Gao C., Wen L., Chen J., Song S. (2013). BiOCl/BiVO_4_ p-n Heterojunction with Enhanced Photocatalytic Activity under Visible-Light Irradiation. J. Phys. Chem. C.

[19] Chang X., Wang T., Peng Z., Zhang J., Li A., Gong J. (2015). Enhanced Surface Reaction Kinetics and Charge Separation of p-n Heterojunction CO_3_O_4_/BiVO_4_ Photoanodes. J. Am. Chem. Soc..

[20] Yu H., Wang J., Wang T., Yu H., Yang J., Liu G., Qiao G., Yang Q., Cheng X. (2017). Scalable colloidal synthesis of uniform Bi_2_S_3_ nanorods as sensitive materials for visible-light photodetectors. CrystEngComm.

[21] Kumar S., Sharma S., Umar A., Kansal S.K. (2016). Bismuth Sulphide (Bi_2_S_3_) Nanotubes as an Efficient Photocatalyst for Methylene Blue Dye Degradation. Nanosci. Nanotechnol. Lett..

[22] Ma D.K., Guan M.L., Liu S.S., Zhang Y.Q., Zhang C.W., He Y.X., Huang S.M. (2012). Controlled synthesis of olive-shaped Bi_2_S_3_/BiVO_4_ microspheres through a limited chemical conversion route and enhanced visible-light-responding photocatalytic activity. Dalton Trans..

[23] Liu C., Li J., Li Y., Li W., Yang Y., Chen Q. (2015). Epitaxial growth of Bi_2_S_3_ nanowires on BiVO_4_ nanostructures for enhancing photoelectrochemical performance. RSC Adv..

[24] Gao X., Wu H.B., Zheng L., Zhong Y., Hu Y., Lou X.W. (2014). Formation of Mesoporous Heterostructured BiVO_4_/Bi_2_S_3_ Hollow Discoids with Enhanced Photoactivity. Angew. Chem..

[25] Obregón S., Colón G. (2013). On the different photocatalytic performance of BiVO_4_ catalysts for Methylene Blue and Rhodamine B degradation. J. Mol. Catal. A Chem..

[26] Zhou X., Ma L., Feng Z. (2015). Synthesis and Characterization of Bismuth Sulfide Nanorods by Solvothermal Route. Chem. Lett..

[27] Zheng Y., Wu J., Duan F., Xie Y. (2007). Gemini Surfactant Directed Preparation and Photocatalysis of m-BiVO_4_ Hierarchical Frameworks. Chem. Lett..

[28] Zhang H., Ji Y., Ma X., Xu J., Yang D. (2003). Long Bi_2_S_3_ nanowires prepared by a simple hydrothermal method. Nanotechnology.

[29] Fu Y., Sun X., Xin W. (2011). BiVO_4_-graphene catalyst and its high photocatalytic performance under visible light irradiation. Mater. Chem. Phys..

[30] Chu W., Tao T., Xu J. (2016). Synthesis, characterization and photocatalytic property of BiOI/Bi_2_S_3_/AOCF composite. New Chem. Mater..

[31] Vu T.H. (2013). Water-Guest Interactions under Clathrate Hydrate Formation Conditions: A Matrix-Isolation Approach. Ph.D. Thesis.

[32] Malakooti R., Cademartiri L., Akçakir Y., Petrov S., Migliori A., Ozin G.A. (2006). Shape-Controlled Bi_2_S_3_ Nanocrystals and Their Plasma Polymerization into Flexible Films. Adv. Mater..

[33] Marton D., Boyd K.J., Al-Bayati A.H., Todorov S.S., Rabalais J.W. (1994). Carbon nitride deposited using energetic species: A two-phase system. Phys. Rev. Lett..

[34] Ou M., Zhong Q., Zhang S., Nie H., Lv Z., Cai W. (2016). Graphene-decorated 3D BiVO_4_ superstructure: Highly reactive (040) facets formation and enhanced visible-light-induced photocatalytic oxidation of NO in gas phase. Appl. Catal. B.

[35] Li D., Wang W., Jiang D., Zheng Y., Li X. (2015). Surfactant-free hydrothermal fabrication of monoclinic BiVO_4_ photocatalyst with oxygen vacancies by copper doping. RSC Adv..

[36] Nair P.K., Huang L., Nair M.T.S., Hu H., Meyers E.A., Zingaro R.A. (1997). Formation of p-type Cu_3_BiS_3_ absorber thin films by annealing chemically deposited Bi_2_S_3_-CuS thin films. J. Mater. Res..

[37] Chen Z., Cao M. (2011). Synthesis, characterization, and hydrophobic properties of Bi_2_S_3_ hierarchical nanostructures. Mater. Res. Bull..

[38] Morgan W.E., Stec W.J., Wazer J.R.V. (1973). Inner-orbital binding-energy shifts of antimony and bismuth compounds. Inorg. Chem..

[39] Lin L., Yang Y., Men L., Wang X., He D., Chai Y., Zhao B., Ghoshroy S., Tang Q. (2013). A highly efficient TiO_2_@ZnO n-p-n heterojunction nanorod photocatalyst. Nanoscale.

[40] Petala A., Bontemps R., Spartatouille A., Frontistis Z., Antonopoulou M., Konstantinou I., Kondarides D.I., Mantzavinos D. (2017). Solar light-induced degradation of ethyl paraben with CuO*_x_*/BiVO_4_: Statistical evaluation of operating factors and transformation by-products. Catal. Today.

[41] Dos Santos W.S., Almeida L.D., Afonso A.S., Rodriguez M., Mesquita J.P., Monteiro D.S., Lca O., Fabris J.D., Pereira M.C. (2016). Photoelectrochemical water oxidation over fibrous and sponge-like BiVO_4_/β-Bi_4_V_2_O_11_ photoanodes fabricated by spray pyrolysis. Appl. Catal. B.

[42] Vogel R., Hoyer P., Weller H. (1994). Quantum-Sized PbS, CdS, Ag_2_S, Sb_2_S_3_, and Bi_2_S_3_ Particles as Sensitizers for Various Nanoporous Wide-Bandgap Semiconductors. J. Phys. Chem..

[43] Wu T., Zhou X., Zhang H., Zhong X. (2010). Bi_2_S_3_ nanostructures: A new photocatalyst. Nano Res..

[44] Bessekhouad Y., Mohammedi M., Trari M. (2002). Hydrogen photoproduction from hydrogen sulfide on Bi_2_S_3_ catalyst. Sol. Energy Mater. Sol. Cells.

[45] Zhao D., Zong W., Fan Z., Fang Y.W., Xiong S., Du M., Wu T., Ji F., Xu X. (2017). Synthesis of carbon-doped nanosheets m-BiVO_4_ with three-dimensional (3D) hierarchical structure by one-step hydrothermal method and evaluation of their high visible-light photocatalytic property. J. Nanopart. Res..

[46] Tan G., Zhang L., Ren H., Wei S., Huang J., Xia A. (2013). Effects of pH on the hierarchical structures and photocatalytic performance of BiVO_4_ powders prepared via the microwave hydrothermal method. ACS Appl. Mater. Interfaces.

[47] Hood M.A., Landfester K., Muñozespí R. (2014). The Role of Residue Acidity on the Stabilization of Vaterite by Amino Acids and Oligopeptides. Cryst. Growth Des..

[48] Rai D., Yui M., Schaef H., Kitamura A. (2010). Thermodynamic Model For BiPO_4_(Cr) And Bi(OH)_3_(Am) Solubility In The Aqueous Na^+^-H^+^-H_2_PO_4_^−^-HPO_4_^2−^-PO4^3−^-OH^−^-Cl^−^-H_2_O System. J. Solut. Chem..

[49] Jiang Y.R., Lin H.P., Chung W.H., Dai Y.M., Lin W.Y., Chen C.C. (2015). Controlled hydrothermal synthesis of BiO*_x_*Cl*_y_*/BiO*_m_*I*_n_* composites exhibiting visible-light photocatalytic degradation of crystal violet. J. Hazard. Mater..

[50] Huang S.T., Jiang Y.R., Chou S.Y., Dai Y.M., Chen C.C. (2014). Synthesis, characterization, photocatalytic activity of visible-light-responsive photocatalysts BiO*_x_*Cl*_y_*/BiO*_m_*Br*_n_* by controlled hydrothermal method. J. Mol. Catal. A Chem..

[51] Ren L., Zhang D., Hao X., Xiao X., Gong J., Wang M., Tong Z. (2016). Synthesis and photocatalytic performance of Bi_2_S_3_/SnS_2_ heterojunction. Funct. Mater. Lett..

[52] Xue C., Yan X., Ding S., Yang G. (2016). Monodisperse Ag-AgBr nanocrystals anchored on one-dimensional TiO_2_ nanotubes with efficient plasmon-assisted photocatalytic performance. RSC Adv..

[53] Tang J., Li D., Feng Z., Tan Z., Ou B. (2013). A Novel AgIO_4_ Semiconductor with Ultrahigh Activity in Photodegradation of Organic Dyes: Insights into the Photosensitization Mechanism. Cheminform.

[54] Lin H.P., Lee W.W., Huang S.T., Chen L.W., Yeh T.W., Fu J.Y., Chen C.C. (2016). Controlled hydrothermal synthesis of PbBiO_2_Br/BiOBr heterojunction with enhanced visible-driven-light photocatalytic activities. J. Mol. Catal. A Chem..

[55] Lin H.P., Chen C.C., Lee W.W., Lai Y.Y., Chen J.Y., Chen Y.Q., Fu J.Y. (2015). Synthesis of a SrFeO_3−*x*_/g-C_3_N_4_ heterojunction with improved visible-light photocatalytic activities in chloramphenicol and crystal violet degradation. RSC Adv..

[56] Yang C.T., Lee W.W., Lin H.P., Dai Y.M., Chi H.T., Chen C.C. (2016). A novel heterojunction photocatalyst, Bi_2_SiO_5_/g-C_3_N_4_: Synthesis, characterization, photocatalytic activity, and mechanism. RSC Adv..

[57] Du M., Xiong S., Wu T., Zhao D., Zhang Q., Fan Z., Zeng Y., Ji F., He Q., Xu X. (2016). Preparation of a Microspherical Silver-Reduced Graphene Oxide-Bismuth Vanadate Composite and Evaluation of Its Photocatalytic Activity. Materials.

